# Prospective randomised trial comparing thermal ablation With laparoscopic Adrenalectomy as an alternatiVE treatment for unilateral asymmetric primary aldosteronism: a protocol for the WAVE trial

**DOI:** 10.1136/bmjopen-2025-111798

**Published:** 2026-03-26

**Authors:** Kate Laycock, Jessica Kearney, Yun-Ni Lee, James Macfarlane, Emily Goodchild, Elisabeth Ng, Antonine Pineau Mitchell, Xilin Wu, Zin Htut, George Goodchild, Deborah Lowe, Nicholas Hilliard, Edmund Godfrey, Nad Qazi, Jose Bastos, Aldons Chua, Razeen Mahroof, August Palma, Aklima Khatun, Chetna Varsani, Daniela Benu, Stephen P Pereira, Mohan Krishnamurthy, Heok Cheow, Teng-Teng Chung, Paul Carroll, Samuel O’Toole, Florian Wernig, Mark Gurnell, M Brown, William M Drake

**Affiliations:** 1 Queen Mary University of London, London, UK; 2 St Bartholomew’s Hospital, London, England, UK; 3 Endocrinology ASO/EASO COM, King’s College Hospital NHS Trust, London, UK; 4 Cambridge University Hospitals NHS Foundation Trust, Cambridge, England, UK; 5 Imperial College London, London, England, UK; 6 Gastroenterology, St Bartholomew’s Hospital, London, England, UK; 7 The Royal London Hospital, London, England, UK; 8 Institute for Liver and Digestive Health, University College London, London, UK; 9 Nuclear Medicine, St Bartholomew’s Hospital, London, England, UK; 10 Nuclear Medicine, Cambridge University Hospitals NHS Foundation Trust, Cambridge, England, UK; 11 Endocrine, University College London, London, England, UK; 12 Endocrine, Guy's and St Thomas’ Hospitals NHS Trust, London, England, UK; 13 Sheffield Teaching Hospitals NHS Foundation Trust, Sheffield, England, UK

**Keywords:** Adrenal disorders, Endocrine tumours, Hypertension

## Abstract

**Introduction:**

Unilateral aldosterone-producing adenomas (>1 cm) and aldosterone-producing nodules (<1 cm) are a common cause of primary aldosteronism (PA) and hypertension. Adrenal surgery can potentially cure or significantly improve the condition. However, fewer than 1% of patients with PA undergo adrenalectomy. For some, this is due to surgical risks, service capacity or patient preference against surgery. In these individuals, thermal ablation may provide an alternative. This paper describes the protocol for the WAVE trial, designed to compare thermal ablation with the current standard, laparoscopic adrenalectomy, in the treatment of unilateral PA.

**Methods and analysis:**

WAVE is a prospective, multicentre, randomised controlled non-inferiority trial comparing thermal ablation (experimental arm) with laparoscopic adrenalectomy (control arm) in the treatment of unilateral PA. 122 participants will be recruited and randomised to thermal ablation or laparoscopic adrenalectomy in a 1.5:1 ratio. Hierarchical co-primary endpoint data considering both biochemical and clinical outcomes will be judged at 6 months. Secondary endpoint data will consider adverse events, length of inpatient stay, patient satisfaction and time to return to activities of daily living. The full protocol is available at ClinicalTrials.gov.

**Ethics and dissemination:**

The protocol was approved by the Bloomsbury Research Ethics Committee (21/LO/0243). The results of the study will be shared with study participants, published in peer-reviewed journals and presented at national/international conferences.

**Trial registration number:**

NCT05405101.

**STRENGTHS AND LIMITATIONS OF THIS STUDY**

- WAVE is a PROBE (Prospective Randomised Open-Label with Blind Endpoint determination) trial that uses international consensus criteria to determine biochemical and clinical outcomes.
- The analysis will report the outcomes of both left-sided (endoscopic) and right-sided (percutaneous) thermal ablation, using radiofrequency or microwave techniques.
- Molecular positron emission tomography imaging is being used pre-ablation and post-ablation to evaluate the extent of ablation of the targeted nodule(s).
- Immunohistochemical and/or molecular (RNA sequencing/quantitative PCR) confirmation of correct targeting of the ablated nodule is possible for left-sided ablations, but not routinely for those on the right.
- WAVE evaluates emerging interventional strategies designed to expand the availability of non-surgical modalities for the management of primary aldosteronism.

## Introduction

 Primary aldosteronism (PA) is the most common cause of secondary hypertension. It is responsible for 5–10% of all hypertension^1 2^ and up to 20–25% of resistant cases.^2^ PA is a high-risk subset of hypertension associated with a twofold higher rate of cardiovascular events and atrial fibrillation relative to age-matched and gender-matched patients with essential hypertension.^3 4^ It is also linked to reduced quality of life.^5^

Aldosterone excess in PA may originate from one or both adrenal glands. Unilateral PA is usually caused by an aldosterone-producing adenoma (APA)/aldosterone-producing nodule (APN) and is conventionally treated with surgical removal of the culprit adrenal gland. While medical therapies such as mineralocorticoid receptor antagonists (MRAs) address aldosterone excess, surgery may be more effective in reducing cardiovascular^6^ and stroke^7^ risk, and improving quality of life.^8^ Furthermore, sufficient MRA dosing to de-suppress renin and mitigate this excess risk is only achieved in one-third of medically treated patients.^3^

Surgery for PA conventionally involves removal of the whole adrenal gland to treat a condition caused by a small (usually <2 cm diameter, and often <1 cm) benign APA/APN. A return to full physical activities takes several weeks and the clinical outcome is uncertain, with only a minority of people stopping anti-hypertensive medication altogether. An alternative intervention to laparoscopic adrenalectomy, and the focus of this trial, is selective thermal ablation (by radiofrequency or microwave) of the identified APA/APN(s). Thermal ablation is a technique in which targeted and directed tissue death can be achieved with precision under image guidance, sparing the normal adrenal gland.

The primary objective of WAVE is to test the hypothesis that thermal ablation (using either microwave or radiofrequency (RFA)) is non-inferior to surgery in the biochemical (and if so, in the clinical) cure of unilateral PA, according to the international consensus primary aldosteronism surgical outcomes (PASO) criteria.^9^

## Methods

This study received ethical approval by the London—Bloomsbury Research Ethics Committee (21/LO/0243, bloomsbury.rec@hra.nhs.uk). The trial registration number is NCT05405101.

### Trial design

WAVE is a prospective, multicentre, randomised controlled non-inferiority trial comparing thermal ablation (experimental arm) with laparoscopic adrenalectomy (control arm; current standard of care) in the treatment of unilateral PA. Adult patients with unilateral PA (proven by adrenal vein sampling and/or molecular imaging) who are candidates for unilateral adrenalectomy, but in whom thermal ablation is also technically feasible, will be recruited. Participants will be randomised in a 1.5:1 fashion to either ablation or surgery. The trial started recruiting in September 2022 and is anticipated to complete recruitment in early 2026. Allowing for 6 months following intervention until collection of primary endpoint data, the trial is expected to complete in mid 2027.

The primary hypothesis of WAVE is that thermal ablation is non-inferior to surgery in the treatment of unilateral PA due to an APA/APN. WAVE is an ‘inferiority non-inferiority study’ design^10^ and draws on experience from other areas of medicine, in which numerically ‘worse’ treatments in terms of efficacy may be deemed ‘non-inferior’ by virtue of other advantages, such as procedural safety, drug toxicity and overall patient acceptability. This approach in clinical trials design requires careful consideration of an appropriate, prespecified non-inferiority margin. This, in turn, is influenced by the severity of the endpoint, the availability of other options in the event of treatment failure and the anticipated benefits of the experimental intervention if adopted into routine clinical practice. Non-inferiority margins in this context are determined by a combination of patients’ views and the opinions of experienced clinicians/investigators in the field. Selected APA thermal ablation cannot be superior to surgical removal of the whole adrenal (which may also harbour additional microscopic foci of autonomous aldosterone production), but is balanced against its less invasive nature. The public and patient involvement in the setting of the non-inferiority margins for WAVE is described in detail in the relevant section.

### Setting

The trial is coordinated at Queen Mary, University of London and involves six participating centres:

1. Barts Health NHS Trust,
2. University College London Hospital NHS Foundation Trust,
3. Imperial College Healthcare NHS Trust,
4. Cambridge University Hospitals NHS Foundation Trust,
5. Guy’s and St Thomas’ NHS Foundation Trust and
6. Sheffield Teaching Hospitals NHS Foundation Trust.

Left-sided ablations will be performed at sites 1, 2 and 4. Right-sided ablations will be performed at sites 1, 3 and 4. Unilateral adrenalectomy may be performed at all sites.

### Study inclusion

122 adults with unilateral PA will be recruited to WAVE. The full inclusion and exclusion criteria are listed in box 1.

Box 1 **. Inclusion and exclusion criteria**

**Inclusion criteria**

Participants must meet all of the following:

- Age >18 years.
- Primary aldosteronism according to international guidelines.^15 16^
  - An elevated aldosterone renin ratio (according to local reference ranges), and at least one of (when measured off confounding medications):
    1. Spontaneous or diuretic-induced hypokalaemia, plasma aldosterone concentration >550 pmol/L and plasma renin activity (PRA) <0.5 nmol/L/hour or direct renin concentration (DRC) <4.2 mU/L.
    2. A positive saline infusion test.
      - 4-hour aldosterone >190 pmol/L on immunoassay (or equivalent, >160 pmol/L on mass spectrometry).
    3. A positive captopril challenge test, either:
      - Failure to suppress 2-hour aldosterone by >30% and persistent suppression of PRA/DRC^15^, or
      - 2-hour aldosterone >300 pmol/L^16^ (by immunoassay or equivalent on alternative assay).

- Unilateral primary aldosteronism (PA), defined by at least one of the following criteria:
  1. Adrenocorticotropic hormone (ACTH)-stimulated adrenal vein sampling (AVS).^17^
    - Selectivity Index (SI) >3, and
    - Lateralisation Index (LI) >4.
  2. Non-ACTH-stimulated AVS.^17^
    - SI >2, and
    - LI >3, and
    - Contralateral Suppression Index <0.5.
  3. Metomidate/para-chloro-2-[^18^F]fluoroethyletomidate (CETO) positron emission tomography (PET)-CT.^18 19^
    - >25% higher PET signal (maximum standardised uptake value) over a focal adrenal macro (>1 cm) or micro (<1 cm) nodule compared with the contralateral adrenal.
  4. Age <35, marked PA, unilateral focal adrenal lesion with normal contralateral gland.^15^
- Radiological abnormality ipsilateral to side of lateralisation, which is:
  1. Benign:
    - Unenhanced CT attenuation <10 Hounsfield units, or
    - Post-contrast CT absolute washout >60%, or
    - Post-contrast CT relative washout >40%, or
    - Signal drop-out on out-of-phase MRI.
  2. Technically amenable to both ablation and surgery (determined at multidisciplinary team (MDT) review). This excluded participants with morbid obesity.
- Able and willing to give informed consent.
- Randomisation approved by MDT.

**Exclusion criteria**

Potential participants will be ineligible to enter the study if they meet any of the following exclusion criteria:

- Absolute contraindication to α-adrenoceptor or β-adrenoceptor antagonist therapy or CT contrast, or
- Contraindication or unwillingness to undergo either surgery or ablation, or
- Inability to withdraw β-adrenoceptor antagonist therapy for 2 weeks, or
- Unwilling to comply with study visit schedule, or
- Pregnancy or unwillingness to undertake secure contraception for the study duration (female participants only), or
- Life-limiting comorbidity (at the discretion of the principal investigator), or
- Clinical and/or biochemical evidence of autonomous cortisol secretion sufficient, in the opinion of the patient’s physician, to mandate a unilateral adrenalectomy independent of autonomous aldosterone secretion, or
- Aldosterone-producing adenoma diameter >2.5 cm.

### Study procedures

**Figure 1:**
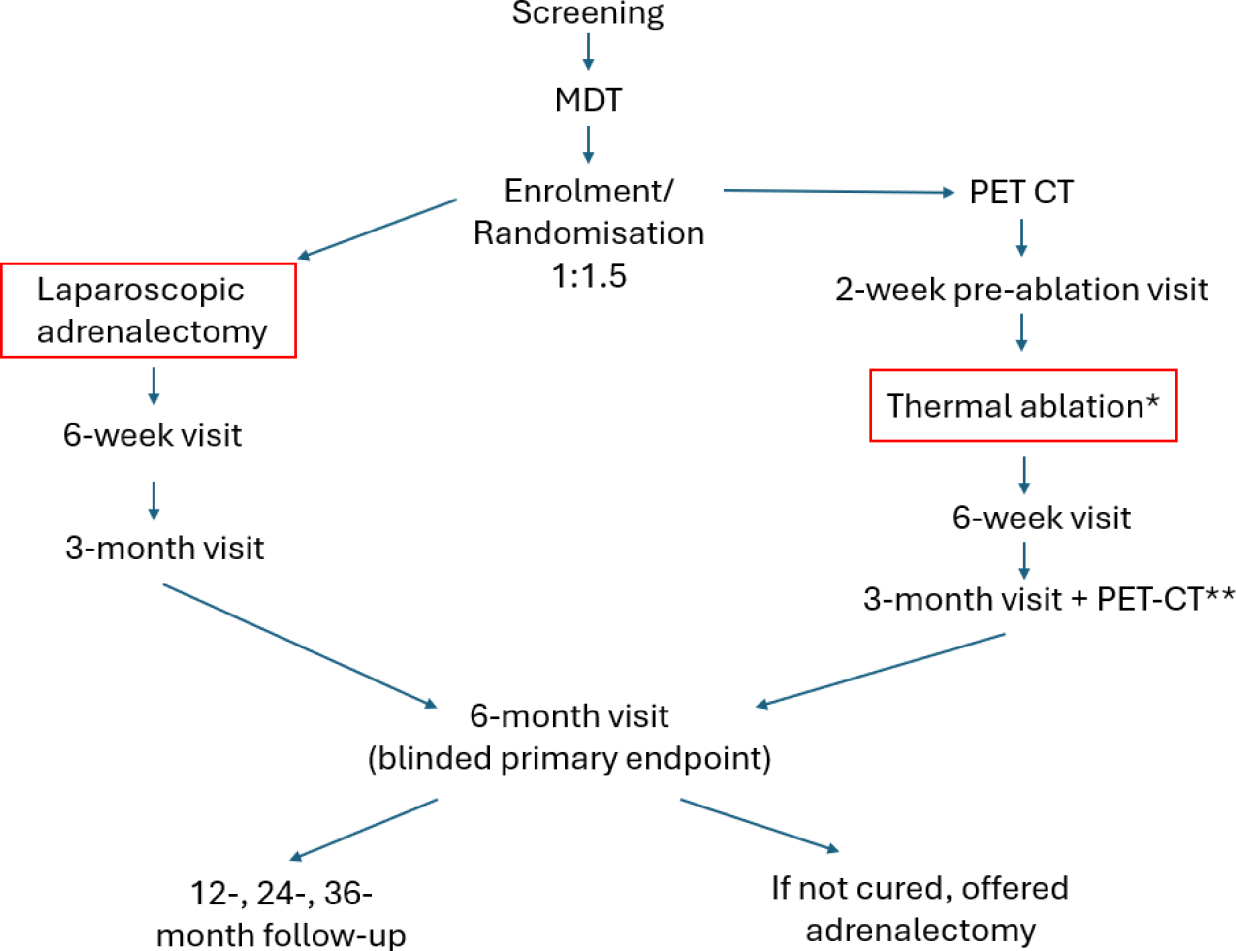
Study timeline following screening, participants are discussed at the MDT and, if deemed suitable, invited for enrolment and randomisation. They will then embark on the surgical or thermal ablation pathway. Patients undergoing thermal ablation require a Metomidate/CETO PET-CT as well as a period of pretreatment with α-adrenoceptor and β-adrenoceptor antagonists prior to intervention. Primary endpoint data are collected at 6 months but patients who reach that end point before the end of the trial will also be seen at 12, 24 and 36 months post-intervention. *Some patients will require two thermal ablations, **the post-ablation PET-CT will occur 3–6 months after the procedure. CETO, fluorine-18-labelled para-chloro-2-fluoroethyl-etomidate; MDT, multidisciplinary team; PET, positron emission tomography.

The timeline for the study is shown in figure 1.

#### Recruitment/screening

Patients with confirmed unilateral PA who express an interest in participating will be identified for recruitment and given a participant information sheet (PIS). Contact details for the trial coordinator are published online to provide an opportunity for patients cared for at other centres to enquire about potential trial participation.

#### Multidisciplinary team meeting

All potential participants will be discussed at the multidisciplinary team (MDT) meeting and eligibility criteria will be scrutinised and recorded. The MDT comprises representatives from all centres and includes clinicians and investigators with the necessary expertise to inform each of the following eligibility criteria:

- Confirm diagnosis of unilateral PA (endocrinologist/hypertension specialist).
- Confirm benign adrenal lesion (radiologist).
- Confirm surgical candidacy (endocrine surgeon).
- Confirm candidacy for percutaneous CT-guided thermal ablation (interventional radiologist).
- Confirm candidacy for endoscopic ultrasound (EUS)-guided RFA (interventional endoscopist).

In patients who have unilateral disease on adrenal vein sampling (AVS) and/or positron emission tomography (PET)-CT, the MDT may exercise discretion to include patients who have not met every component of the Endocrine Society’s consensus guidelines for the diagnosis of PA but where the diagnosis is universally accepted by all principal investigators (PIs). For example, a patient with hypokalaemia with a fully suppressed renin (in the absence of a beta-blocker) but where aldosterone does not quite achieve 550 pmol/L and a confirmatory test has not been performed would be eligible for inclusion.

#### Enrolment visit and randomisation

Following confirmation of eligibility by the MDT, participants will attend for enrolment. Informed written consent will be obtained by a Good Clinical Practice-trained member of the clinical research team. The consent form can be found in online supplemental material 1. No incentives were provided to participants for taking part in the study. Baseline clinical data, blood and urine samples will be collected and 24 hours ambulatory blood pressure monitoring (24hABPM) will be performed. Quality of life (EuroQol 5-Dimension 5-Level questionnaire (EQ-5D-5L), 36-Item Short Form Health Survey (SF-36)) questionnaires will be completed.

Patients will be randomised using a minimisation technique in a 1.5:1 ratio to ablation or surgery, stratified according to site, laterality (left vs right), gender (male vs female) and ethnicity (non-black vs black) by a computerised system operated by King’s College London Clinical Trial Unit (KCTU). Due to the nature of the interventions, blinding or sham procedures are not appropriate.

If randomised to thermal ablation, patients whose PA was lateralised by AVS will undergo molecular adrenal imaging in order to localise the ablation target. All patients recruited from Site 1 will be invited to have a non-compulsory cardiovascular magnetic resonance scan (CMR).

#### 2-week pre-ablation visit (thermal ablation group only)

For patients undergoing thermal ablation, α-adrenoceptor and β-adrenoceptor antagonists will be initiated, and subsequently titrated, 2 weeks prior. This is to mitigate the risk of a procedural hypertensive emergency due to adrenomedullary stimulation. The exact choice of agent will be at the discretion of the site PI, with suggested starting doses of doxazosin 2 mg two times a day and bisoprolol 2.5 mg once daily. After 1 week, these will be up-titrated aiming for a dose of doxazosin of 4 mg two times a day and bisoprolol 5 mg once daily if tolerated. Depending on blood pressure (BP) control, additional alteration of concomitant anti-hypertensive therapy may be required, with the choice at the discretion of the PI.

#### Ablation

Thermal ablation will be delivered using CT-guidance for right-sided APAs and EUS-guidance for left-sided APAs. Procedures may be performed under general anaesthetic or sedation with BP monitoring, invasive or non-invasive, at the discretion of the supervising anaesthetist.

Prior to thermal ablation, if feasible, research fine needle biopsies of the APA and adjacent normal adrenal tissue will be taken for immunohistochemistry, gene expression and somatic mutation analysis. RFA is performed using a 5 or 10 mm 19G STARMed (Gyeonggi-go, Korea) ablation catheter. The stomach is able to self-seal following ablation. For right-sided APAs, the intention is to ablate from a retroperitoneal approach avoiding lung, kidney and liver. However, in selected cases, a transhepatic approach may be used where required in order to avoid the lung and the risk of pneumothorax.

During the ablation, the following details will be recorded: duration of procedure, medications administered, maximum BP as well as the number and duration of treatments (‘burns’). Plasma metanephrines will be taken three times on the day of the thermal ablation procedure (pre-anaesthetic, post-anaesthetic/sedation but prior to insertion of the ablation catheter and during ablation). Additional samples for plasma metanephrines will be collected if there is a clinically concerning rise in BP and/or heart rate during or immediately after the procedure.

A specific potential risk of APA thermal ablation (which is not shared in laparoscopic adrenalectomy) is stimulation of catecholamine release from the adjacent adrenal medulla which can result in significant hypertension and tachycardia akin to a ‘phaeochromocytoma crisis’. In order to mitigate against this risk in WAVE, patients will be alpha and beta blocked pre-ablation and have BP monitoring during the procedure with necessary pharmacological agents at hand if required. Standard safety considerations apply to the risk of bleeding and pneumothorax.

Following thermal ablation, participants will be monitored according to local protocol and can be discharged on the same day. The following day, participants will return for a clinical review and protocol-specified blood tests. Antihypertensive medications will be optimised accordingly with a preference for classes that do not significantly interfere with measurements of renin and aldosterone.

#### Surgery

Laparoscopic adrenalectomy will, in general, be carried out at the enrolling site, according to local standard operating procedure (SOP), but may, if the patient wishes, be carried out at the referring centre. Alternatively, should the patient or local PI feel it is more appropriate, then it may be carried out elsewhere.

During surgery, the following details will be recorded: duration of procedure, medications administered and maximum BP. Plasma metanephrines will be taken three times on the day of surgery (pre-anaesthetic, post-anaesthetic but pre-procedure and during the surgery). Both APA and adjacent normal adrenal tissue will be collected for immunohistochemical analysis, gene expression and somatic mutation analysis.

On postoperative day 1, participants will be seen for a clinical review and protocol-specified blood tests. Antihypertensive medications (if required, with a preference for those that do not interfere with measurements of renin and aldosterone) will be optimised.

#### Second ablation

A small subset of patients will be offered a second ablation following MDT discussion. This would not usually be considered if the nodule is <2 cm in maximum diameter. For patients with more than one left-sided adrenal nodule, if the repeat PET-CT shows that thermal ablation did not target the likely APA and instead another adrenal nodule was ablated, a second ablation procedure will be recommended.

#### Post-intervention visits at 6 weeks and 3, 6, 12, 24 and 36 months

Clinic and home BP measurements will be recorded at each visit. Medication history will be reviewed, and antihypertensive medication titrated according to BP.

Secondary outcome data, including patient satisfaction and date of return to usual activities of daily living, will be completed at 6 weeks.

Patients in the thermal ablation group will have a post-ablation Metomidate or CETO PET-CT scan at 3 months to help assess whether there is any residual APA following ablation. Patients can be offered a second ablation if the criteria described previously apply.

Primary outcome data will be collected at the 6-month visit. All interfering medications that could affect aldosterone or renin will be stopped prior to the appointment. If the aldosterone renin ratio (ARR) is elevated, a confirmatory test (saline infusion test or captopril challenge test at the local PI’s discretion) will be performed. 24hABPM and quality of life questionnaires (EQ-5D 5L, SF-36) will be collected at this visit. For patients who had a CMR at enrolment, the scan will be repeated.

While the trial is ongoing, patients who reach the 12-month, 24-month and 36-month mark post-intervention will be scheduled for a study visit.

#### Adrenalectomy post-ablation

Participants who undergo thermal ablation and do not experience complete biochemical success by PASO criteria at the 6-month time point may be offered a unilateral adrenalectomy as standard treatment for lateralised PA. This will only be considered after MDT review of the post-ablation PET-CT. If surgery occurs during the course of the trial, 6-month post-surgical follow-up will be recorded as for other surgical patients but will not constitute the primary outcome data.

#### End of study visit

The end of the study is defined as the final study visit of the final participant. This will be the 6-month post-intervention visit of the final participant.

### Ablation therapy

Participants randomised to thermal ablation will undergo assessment by the treating clinician (endoscopist for left-sided APA, interventional radiologists for right-sided APA). RFA will be used for left-sided APAs, guided by endoscopic ultrasound. For right-sided APAs, microwave or RFA will be selected at the discretion of the interventional radiologist, based on location of the APA and their expert opinion on which method is most likely to be effective.

### Outcomes

WAVE is a PROBE (Prospective Randomised Open-Label with Blind Endpoint determination) trial. Both primary endpoints (biochemical indices and electronic BP monitor downloads) will be entered onto the electronic case report form (eCRF) by personnel blinded to the intervention.

#### Primary endpoint

Hierarchical primary endpoints will be adjudged at 6 months post intervention (for patients who have had two ablations, this will be 6 months post second intervention) and compared with the enrolment data:

1. Complete biochemical cure of PA, defined (while off medications that might alter serum potassium, renin or aldosterone) by both:
  - Normalisation of serum potassium, and
  - Normalisation of ARR, or
  - Elevated ARR and
    - Baseline plasma aldosterone concentration <190 pmol/L on immunoassay (<160 pmol/L on mass spectrometry), or
    - Normal confirmatory test (as defined in the inclusion criteria).
2. Complete clinical cure of PA, defined as normotension without antihypertensive medication.

These criteria have been defined in the international consensus PASO statement,^9^ which has become the established yardstick by which outcomes following unilateral adrenalectomy are judged. In this, normotension is defined, in accordance with the European Society of Hypertension guidelines,^11^ as <140/90 in the office, <135/85 at home or daytime ambulatory monitoring and <130/80 for 24hABPM. The preferred method of assessment of BP will be via 24hABPM, with home BP allowable for subjects unable to perform 24hABPM.

In addition to the primary endpoint data, other exploratory analyses will include predictors of clinical success such as age, duration of hypertension and the dose and number of antihypertensives, in line with other published series.

#### Secondary endpoints

Frequency and severity of adverse events will be reported throughout the study period and be classified by system, seriousness, causal relationship and expectedness according to the Common Terminology Criteria for Adverse Events V.5.0. Prespecified post-intervention safety aspects will be systematically evaluated and, where appropriate, compared between the two interventions, specifically anaemia, renal dysfunction and electrolyte abnormalities, liver dysfunction, pancreatitis and procedure-related hypertensive emergencies.

Length of inpatient stay will be recorded at the 6-week visit alongside patient satisfaction (Freiburg Index of patient satisfaction^12^) and duration to return to activities of daily living.

The anatomical efficacy of ablation will be assessed in the ablation group using molecular adrenal imaging appearances 3–6 months post ablation.

The presence of partial biochemical and clinical cure, as determined by the international consensus PASO statement,^9^ will be assessed as a secondary endpoint, while complete cure is a primary endpoint.

Post-intervention analysis of tissue samples will include histological grading according to the HISTALDO consensus^13^ (surgical specimens only), immunohistochemistry, studies of gene expression and genotyping for somatic mutations.

### Data management

#### Electronic case report form and randomisation

A web-based eCRF has been designed using the InferMed Macro 4 system alongside a bespoke KCTU randomisation system. Both were created in collaboration with the trial analyst and are maintained by the KCTU.

#### Confidentiality

Information related to participants will be kept confidential and managed in accordance with the Data Protection Act, General Data Protection Regulation, NHS Caldecott Principles, the UK Policy Framework for Health and Social Care Research and the conditions of the Research Ethics Committee (REC) favourable opinion.

Research data will be fully anonymised within the eCRF, which will not contain any patient identifiable details. The patient identification list and hard CRFs, which will contain PID, will be securely stored at each site in accordance with relevant organisational data protection SOPs and legislation.

#### Data sharing

Clinical trial data may be made available to qualified academic researchers on approval by the study management committee and completion of appropriate data-sharing and transfer agreements. Data requests must include a clear rationale, the relevance of the proposed research and a description of the hypothesis, research methodology, statistical analysis plan and intended publication plan.

## Planned analysis

### Sample size calculation

The initial sample size calculation for the study was set at 110 participants, with equal numbers of individuals in the surgical and the ablation arm. However, following a revision in the study protocol, the total sample size has been increased to 122 participants. This adjustment accounts for potential withdrawals, with the final analysis set expected to include 110 participants.

The overriding question is to compare thermal ablation with surgery in the management of unilateral PA. However, the ablation procedures on each side are distinct. On the right, fewer nodules are technically amenable to ablation because of their proximity to the inferior vena cava (IVC). This is due to a ‘heat sink’ effect that accompanies the delivery of RFA. The high volume of blood flowing through the IVC rapidly conducts heat away, limiting the effectiveness of the procedure. Additionally, the longer track from the skin to the nodule means that organs, such as the kidney, may obstruct access to the nodule; and in some patients the risk of pneumothorax is unacceptably high. In contrast, endoscopic left-sided ablations do not encounter such issues. Moreover, the recent FABULAS trial has contributed to local expertise in endoscopic ablations. For this reason, the analysis will first consider left-sided APAs and then all APAs. The balance between the two treatment arms has been updated from 1:1 to 1.5:1 (prior to any collection of primary endpoint data), with 66 participants in the ablation arm and 44 in the surgical arm. The sample size for each arm and laterality is shown in table 1.

**Table 1:** Sample size for each arm and laterality

Arm	Ablation		Surgery		
Laterality	Right	Left	Right	Left	
Participants (n)	16	50	11	33	
Total	66		44		110

A total of 110 participants is required for analysis. This includes 66 in the ablation arm (16 right and 50 left) and 44 in the surgical arm (11 right and 33 left). To account for participant withdrawals and occasional technical/safety issues (eg, the splenic vein lying between the stomach and APA, rendering the procedure not possible without undue hazard), the recruitment target is 122.

Endoscopic thermal ablation has already been assessed in the FABULAS proof-of-concept study^14^ and its proven low morbidity, without the need for a full general anaesthetic, validates a high non-inferiority margin to justify consideration in preference to surgery. A −45% margin, though wide, is deemed sufficient to recommend ablation in the future to the many patients, identified during two rounds of patient and public involvement, who would not consider surgery, but wish for a better than 50:50 option of stopping or reducing medicines; while a −30% margin will justify ablation in patients interested in a better than 2:1 margin of avoiding surgery as an initial intervention.

For the first limb of the hierarchical primary outcome, the required sample size was calculated assuming a biochemical success rate of 90% in the surgical group (as observed in the MATCH trial) and 70% in the ablation group (based on data from the FABULAS trial). On this basis, 50 left-side ablation patients and 33 left-side surgical patients are needed to demonstrate non-inferiority (defined as a non-inferiority limit of −45%) with 90% power and a 5% type 2 error rate.

For the second limb of the hierarchical primary outcome, a biochemical success rate of 90% in the surgical group and 65% in the ablation group was assumed. A sample size of 66 ablation patients (right and left) and 44 surgical patients will be required to demonstrate non-inferiority (defined as a non-inferiority limit of −45%) with 85% power and a 5% type 2 error rate.

For the third limb of the hierarchical primary outcome, a complete clinical success rate of 40% in the surgical group (30% in MATCH) and 30% in the ablation group (14% in FABULAS) was assumed. The more optimistic expectation of clinical success in WAVE in the surgical arm reflects the different demographic and requirement for a visible adenoma on cross-sectional imaging. The more optimistic expectation of the ablation group in WAVE reflects the phase one nature of FABULAS and considerable expertise acquired during that study. A sample size of 66 ablation patients (right and left) and 44 surgical patients will be required to demonstrate non-inferiority (defined as a non-inferiority limit of −33.3%) with 80% power.

### Statistical analysis

Hierarchical primary endpoints will be judged at 6 months post intervention and compared with the enrolment criteria data. In this context, the hierarchical endpoint refers to the sequence of primary outcomes that must be assessed in order, with each step building on the previous one. If the first endpoint is not met, then no other endpoints will be assessed. Hierarchical analysis testing will be applied as follows:

1. Complete biochemical cure for left ablation versus left surgery (positive if non-inferiority (NI) margin <45%).
2. Complete biochemical cure for all ablation versus all surgery (positive if NI margin <45%).
3. Complete clinical cure for all ablation versus all surgery (positive if NI margin <33%).

The first limb of the hierarchy will be tested if sufficient patients have been recruited on the left side. The second and third limbs will be tested if enough patient have been recruited in all groups. Each limb will be considered successful if prespecified numerical thresholds are met, as set out in the international PASO consensus and expanded in Section 4.6.1.

Secondary hypotheses will be tested using Fisher’s exact test or analysis of variance, as appropriate, with a focus on seeking superiority. In cases where superiority is not observed, non-inferiority testing will be applied to either continuous variables or proportions.

## Patient and public involvement

Patient involvement has been integral to the conception and design of this trial, with the goal of developing a less invasive alternative to surgery for treating PA. Insights from patients who have undergone ablation during the safety trial FABULAS,^14^ along with input from patient representatives on the Trial Steering Committee, have played a key role in shaping the WAVE protocol and PIS.

To better understand patient perspectives on ablation, a dedicated focus group was conducted, consisting of nine patients with PA with direct experience of clinical trials (specifically MATCH and FABULAS) and PA treatment. The patients expressed a clear preference for the ‘adrenal-sparing’ ablation method, highlighting, in addition to its less invasive nature to surgery, their desire to avoid ‘losing’ an entire adrenal gland through surgery. The possibility of a second ablation in routine practice and the availability of a complete adrenalectomy if ablation was unsuccessful were also key themes to emerge.

To gather broader input, a multinational survey was developed to include a cohort of 181 patients with PA, the majority of whom had no direct connection to the research team. Only 4% said they would not consider ablation as an alternative or preliminary step before surgery. 79% of patients were willing to accept an ablation success rate of ≤75% relative to surgery, with 12% even accepting a ≤25% relative success rate. These views, together with detailed discussion among the research team and clinicians experienced in the management of PA, informed the NI margins chosen for WAVE.

## Ethical considerations

Ethical approval was gained from the local REC and the study conducted in accordance with the UK Policy Framework for Health and Social Care Research and the World Medical Association Declaration of Helsinki (1996). Participation in the trial is voluntary with potential subjects being identified from patients in whom unilateral PA has already been diagnosed and would otherwise be referred for surgery as standard of care. Informed consent is obtained only after sufficient time has elapsed following initial approach and provision of the PIS (minimum 24 hours). Participant confidentiality is maintained throughout.

Adverse events will be recorded in the participants’ documents. Serious adverse events are reported to the sponsor within 24 hours and REC within 15 days if ‘related’ and ‘unexpected’. Annual progress reports are sent to the REC. The Trial Steering Committee and Data Monitoring Committee also receive regular safety reports.

The trial is sponsored by Queen Mary, University of London, who review and approve all protocols, amendments and trial procedures.

## Supplementary material

10.1136/bmjopen-2025-111798 online supplemental file 1
