# Supplementary material for: Prospective randomised trial comparing thermal ablation With laparoscopic Adrenalectomy as an alternatiVE treatment for unilateral asymmetric primary aldosteronism: a protocol for the WAVE trial
Source: BMJ Open. 2026 Mar 26;16(3):e111798. doi: 10.1136/bmjopen-2025-111798 (PMC13034237; doi:10.1136/bmjopen-2025-111798)
Supplement: online supplemental file 1 [file bmjopen-16-3-s001.doc]

Centre Number: Study Number: Participant Identification Number for this trial:

**CONSENT FORM**

Title of Project: **WAVE STUDY (WITH CMR SUB-STUDY)**

A prospective randomised trial comparing thermal ablation **W**ith laparoscopic **A**drenalectomy as an alternati**VE** treatment for unilateral asymmetric primary aldosteronism

Principal Investigators: Prof Morris Brown, Prof William Drake

Please initial box

| 1 | I confirm that I have read the information sheet dated.................... (Version............) for the above study. I have had the opportunity to consider the information, ask questions and have had these answered satisfactorily. |  |
| --- | --- | --- |
| 2 | I understand that my participation is voluntary and that I am free to withdraw at any time without giving any reason, without my medical care or legal rights being affected. |  |
| 3 | I understand that relevant sections of my medical notes and data collected during the study, may be looked at by individuals from where it is relevant to my taking part in this research. I give permission for these individuals to have access to my records. |  |
| 4 | I understand that the information collected about me will be used to support other research in the future and may be shared anonymously with other researchers. |  |
| 5 | I agree to have the additional blood samples for future ethically approved research. |  |
| 6 | I agree to have my adrenal tissue analysed and have read and understood the information sheet |  |
| 7 | I agree to have additional CMR scans as part of the sub-study |  |
| 8 | I agree to take part in the above study |  |

Name of Participant Date Signature

Person taking consent Date Signature
