# Supplementary material for: Prospective randomised trial comparing thermal ablation With laparoscopic Adrenalectomy as an alternatiVE treatment for unilateral asymmetric primary aldosteronism: a protocol for the WAVE trial
Source: BMJ Open. 2026 Mar 26;16(3):e111798. doi: 10.1136/bmjopen-2025-111798 (PMC13034237; doi:10.1136/bmjopen-2025-111798)
Supplement: Supplementary file 2 [file bmjopen-2025-111798.reviewer_comments.pdf]

PEER REVIEW HISTORY

BMJ Open publishes all reviews undertaken for accepted manuscripts. Reviewers are asked to complete a checklist review form and are provided with free text boxes to elaborate on their assessment. These free text comments are reproduced below.

ARTICLE DETAILS

Title (Provisional)

Protocol for the WAVE trial - a prospective randomised trial comparing thermal ablation With laparoscopic Adrenalectomy as an alternatiVE treatment for unilateral asymmetric primary aldosteronism

Authors

Laycock, Kate; Kearney, Jessica; Lee, Yun-Ni; Macfarlane, James; Goodchild, Emily; Ng, Elisabeth; Pineau Mitchell, Antonine; Wu, Xilin; Htut, Zin; Goodchild, George; Lowe, Deborah; Hilliard, Nicholas; Godfrey, Edmund; Qazi, Nad; Bastos, Jose; Chua, Aldons; Mahroof, Razeen; Palma, August; Khatun, Aklima; Varsani, Chetna; Benu, Daniela; Pereira, Stephen P; Krishnamurthy, Mohan; Cheow, Heok; Chung, Teng-Teng; Carroll, Paul; O'Toole, Samuel; Wernig, Florian; Gurnell, Mark; Brown, M; Drake, William M

VERSION 1 - REVIEW

**Reviewer**	**1**
**Name**	**Parameswaran, Rajeev**
**Affiliation**	**Department of Endocrine Surgery, National University Hospital**
**Date**	**02-Dec-2025**
**COI**	**None**

Laycock et al propose a protocol for WAVE trial comparing laparoscopic adrenalectomy versus thermal ablation as treatment modalities for unilateral asymmetric primary aldosteronism. The proposal is an extension of the FABULAS study aimed to assess the efficacy of intervention on biochemical and clinical parameters, along with adverse events. The study proposal is clear and well-structured and will be a useful study in the management of PA.

There are some minor issues that needs clarification.

1. What is the diameter of the probes used for the RFA? Using a 10mm probe transgastrically would leave a 10mm defect in the stomach? How would this defect be closed post ablation?

2. The authors report that Thermal ablation will be delivered using CT-guidance for right-sided APAs. What approach will be used for the right side- transhepatic or retroperitoneal? Supine or lateral?

3. What about morbidly obese patients with PA? Will they be included or excluded?

4. The interventionalists may be very experienced but there is no mention of stopping the ablation in adverse events such as pneumothorax or bleeding. This needs to be highlighted as safety of patient is paramount.

5. Why the need for 2nd ablation after failed 1st ablation and not proceed direct to adrenalectomy?

6. Duration of hypertension, dose and number of antihypertensives may have an impact on post-intervention outcomes. This needs to be compared between the 2 groups, else the cure of hypertension outcomes may be different.

VERSION 1 - AUTHOR RESPONSE

Reviewer 1:

1. What is the diameter of the probes used for the RFA? Using a 10mm probe transgastrically would leave a 10mm defect in the stomach? How would this defect be closed post ablation?
Apologies if this was not immediately clear. RFA is performed using a 5 or 10 mm 19G STARMed (Gyeonggi-go, Korea) ablation catheter. The outer diameter is 1.1mm. 5/10mm is the **length** of the ablation probe. The stomach is able to self-seal following perforation with this needle.

2. The authors report that Thermal ablation will be delivered using CT-guidance for right-sided APAs. What approach will be used for the right side- transhepatic or retroperitoneal? Supine or lateral?
The intention for this study is to aim to ablate from a retroperitoneal approach avoiding lung, kidney, liver. However the transhepatic approach is well documented and this has been required on 2 occasions so far because of difficulty in access from the retroperitoneal approach at the time of ablation due to interposition of lung. CT guided ablation has been performed in the lateral (right side down approach to aim to exclude the lung) and also semi prone approach.

3. What about morbidly obese patients with PA? Will they be included or excluded?
Only patients who were considered safe for surgical intervention were recruited, this meant no patients with morbid obesity were selected.

4. The interventionalists may be very experienced but there is no mention of stopping the ablation in adverse events such as pneumothorax or bleeding. This needs to be highlighted as safety of patient is paramount.
Thank you for this important consideration. Standard safety considerations apply as for other ablation procedures. The main reason to stop an ablation is hypertensive crisis. Significant bleeding is uncommon during the ablation itself and typically occurs after needle withdrawal. A pneumothorax is managed clinically and, if the patient is stable, is not a reason to halt the procedure—just as we would continue when ablating a lung lesion even if a pneumothorax

occurs.

5. Why the need for 2nd ablation after failed 1st ablation and not proceed direct to adrenalectomy?
Experienced ablationists do not consider it feasible to ablate nodules >2cm in one sitting. For this reason, participants with a nodule 2-2.5cm are considered for a second ablation.
It is common for patients with APAs to have additional non-secretory nodules. For patients with multiple nodules in whom the post-ablation PET CT demonstrated that the aldosterone secreting adenoma had not been ablated, they are permitted to have a second ablation. For these cases, failure to ablate the APA is not considered to be failure of the procedure itself (rather an issue with the imaging) and so a second ablation is appropriate.

6. Duration of hypertension, dose and number of antihypertensives may have an impact on post-intervention outcomes. This needs to be compared between the 2 groups, else the cure of hypertension outcomes may be different.
Thank you for this comment. These parameters are captured on the eCRF and will certainly be considered in multivariate analysis. Patients will be randomised using a minimisation technique to ablation or surgery, stratified according to site, laterality (left vs right), gender (male vs female) and ethnicity (non-black vs black).

VERSION 2 - REVIEW

**Reviewer**	**1**
**Name**	**Parameswaran, Rajeev**
**Affiliation**	**Department of Endocrine Surgery, National University Hospital**
**Date**	**29-Jan-2026**
**COI**	

The authors have addressed the concerns raised. Wishing the authors a successful trial and await the outcomes of the study in course of time.
